# Human dimensions of climate change: the vulnerability of small farmers in the Amazon

**DOI:** 10.1098/rstb.2007.0025

**Published:** 2008-02-11

**Authors:** Eduardo S Brondizio, Emilio F Moran

**Affiliations:** 1 Department of Anthropology, Indiana UniversityBloomington, Indiana 47405, USA; 2 Anthropological Center for Training and Research on Global Environmental Change, Indiana UniversityBloomington, Indiana 47405, USA; 3 Center for the Study of Institutions, Population and Environmental Change, Indiana UniversityBloomington, Indiana 47406, USA

**Keywords:** human adaptation, Amazon, human–climate interaction, small farmers, fire-drought, ENSO

## Abstract

This paper argues for a twofold perspective on human adaptation to climate change in the Amazon. First, we need to understand the processes that mediate perceptions of environmental change and the behavioural responses at the levels of the individual and the local population. Second, we should take into account the process of production and dissemination of global and national climate information and models to regional and local populations, especially small farmers. We discuss the sociocultural and environmental diversity of small farmers in the Amazon and their susceptibility to climate change associated with drought, flooding and accidental fire. Using survey, ethnographic and archival data from study areas in the state of Pará, we discuss farmers' sources of knowledge and long-term memory of climatic events, drought and accidental fire; their sources of climate information; their responses to drought and fire events and the impact of changing rainfall patterns on land use. We highlight the challenges of adaptation to climate change created by the influence of migration and family turnover on collective action and memory, the mismatch of scales used to monitor and disseminate climate data and the lack of extension services to translate large-scale forecasts to local needs. We found that for most farmers, memories of extended drought tend to decrease significantly after 3 years. Over 50% of the farmers interviewed in 2002 did not remember as significant the El Niño Southern Oscillation (ENSO) drought of 1997/1998. This helps explain why approximately 40% of the farmers have not changed their land-use behaviours in the face of the strongest ENSO event of the twentieth century.

## 1. Introduction

**Figure 1:**
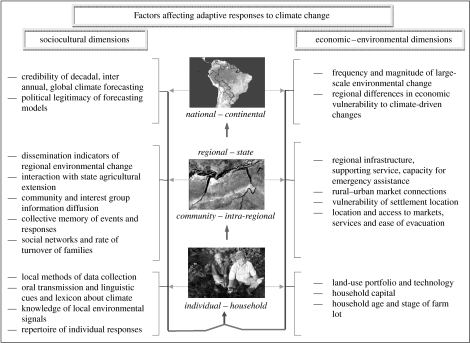
Conceptual framework: factors affecting adaptive responses to climate change in a multilevel perspective.

Why do local populations in the Amazon take different approaches to adapt to climate change than those suggested by outside experts? We argue that two sets of factors are influencing their adaptive strategies: culture and information. First, we need to understand the processes that mediate the perception of environmental change and the differential behavioural adaptations at the levels of the individual and the local population. In this context, adaptation to change and the degree of vulnerability of small farmers are functions of culture (e.g. repertoire of responses, language cues, knowledge of environmental signals), society and economics (e.g. social networks, supporting services, infrastructure) and the environment (e.g. spatial pattern of land cover, vegetation phenology, topography, rainfall distribution). Second, we should take into account the interlinkages of global and national climate information and prognostic models and their dissemination to and interpretation by regional and local populations. We found that for most farmers, memories of extended drought tend to decrease significantly after 3 years. Over 50% of the farmers interviewed in 2002 did not remember as significant the El Niño Southern Oscillation (ENSO) drought of 1997/1998, one of the strongest on record. This helps explain why approximately 40% of the farmers have not changed their land-use behaviours in the face of the strongest ENSO event of the twentieth century. Adaptation to climate change depends on forms of institutional arrangements that facilitate human activities within and across levels (Cash *et al*. 2006). This conceptual framework places farmers' adaptations within a local cultural and environmental setting that includes climate change knowledge (figure 1).

The diversity of an economic portfolio and its use of technology can give greater flexibility to individuals for adjusting to changes. In this sense, many of the world's small farmers have an advantage over large, monocropping farmers when climatic change makes an area inappropriate for one crop. Changing to another crop may be difficult for the larger operator due to the lack of knowledge, having to change large-scale technology for a new crop and the risk of being worse off. Small farmers, who commonly plant a diversity of crops, are likely to see impact in some but not all their crops and can change the composition. However, as illustrated below, small farmers in the Amazon have acute technological limitations (over 90% depend on manual labour and simple tools), little access to extension services and market disadvantages, all of which limit their ability to curb the use of burning, expand construction of firebreaks or invest in more intensive forms of land use.

Amazonian rural populations are diverse and dynamic, varying in their history and time in the region, knowledge of and dependency on the environment, land-use portfolio, cultural ties to the region and density of social networks. They are strongly shaped by cycles of migratory flows, global commodity markets and their interconnection to large-scale infrastructure and environmental systems. Small farming populations in the region are particularly susceptible to three interrelated environmental conditions associated with climate change: extended drought; variation and duration of floods; and spread of accidental fire. These conditions directly affect their economic activities, land uses, livelihoods, residential patterns, dependencies on social networks and exposure to diseases (table 1). The level of intensity and duration of climate anomalies, such as extended drought, affect the vulnerability of small farmers in terms of food security, credit debt, health problems and, in some cases, homelessness and social violence.

We focus our attention in this paper to areas in Amazônia, which have undergone colonization since the 1960s, including farm settlement areas and rural communities along the Transamazon Highway (BR-230) between Altamira and Medicilândia, and the BR-163 highway between Rurópolis and Santarém, both in the state of Pará. Research along the Br-230 has been carried out since 1972, by our team, with major surveys conducted in 1997/1998, 2002 and 2005 using a stratified random sample of arriving cohorts of settlers. The properties chosen for sampling were based on when they began to deforest, leading to membership in a cohort, from each of which a random sample was drawn. Our study began in 2001 along the BR-163 and Santarém, with a major survey of households in 2002 and 2003 derived randomly from a spatially gridded map over all major roads representing the migration waves that took place in the past (1930s, 1950s and 1970s; see discussion of methodology in Moran *et al*. (2005)). This dataset is complemented by ethnographic and land-use data on fire use, accidental fire and flooding collected among riverine and flood plain populations in the region of Santarém. We present information on their sources of knowledge and long-term memories of ENSO, drought and accidental fire; their sources of climate information; their responses to climatic and fire events and the impact of changing rainfall patterns on land use and settlement. Our sample of farm household survey includes 271 and 171 households in the Br-163 and Br-230 regions, respectively.

In analysing these patterns, we pay particular attention to the conditions undermining adaptive responses of small farmers to climate change, because they are more at risk than others whose livelihoods are less dependent on climate. The conditions include high rates of migration, frequent family replacement and lot turnover, community breakdown; interspersion of land-use areas and fire-prone vegetation; and lack of access to basic infrastructure and extension services. We also argue that a mismatch of scales exists between the climate information and the farmers' scope of operation, increasing the lack of credibility of information disseminated through popular media.

In order to address these issues, in §2 we discuss the foundations of our conceptual framework. The proposed framework places farmers' adaptations to climate change within a local cultural and environmental setting as part of a nested system of climate change knowledge and dissemination (figure 1).

## 2. Conceptual approach

Much previous work on adapting to climate change ignores the context in which people must evaluate the risks of changing their livelihood strategies or the risks of avoiding change. New adaptive strategies to climate change do not appear de novo and in a vacuum: actors decide whether the new experience is comparable with anything they have experienced or culturally known and classified in the past. If yes, the adaptive behaviour will probably repeat the previously used, proved scripts. If no, differences in the new experience will trigger more complex assessment to see whether the new experience can be fitted to the existing cultural categories and behaviour or requires more contextual assessment. If the latter is the case, the person/family must evaluate whether the benefits of producing new scripts and routines outweigh the costs, whether risk and uncertainty are increased and how large the pay-off will be if the new routine is useful versus potential failure of a well-tried routine. These risks are assessed depending on the constraints of the individual as a member of a household and a community, and the external constraints he/she faces.

To understand human dimensions of climate change, we need to begin by examining the adaptive mechanisms of human populations to environmental change, the differential responses to the magnitude and the frequency of perceived and actual changes, and the differences between adaptive responses at the individual level and those visible at the population level. Adaptive responses to environmental change are mediated by multiple factors, for example, perception of change in cultural and linguistic dimensions, such as whether people have experienced that type of change, whether it is easily understood and interpreted by existing cues and an appropriate lexicon and whether there is a repertoire of responses to that specific change or to a family of similar changes. If flooding is a regular occurrence in a region, established residents are more likely to have a specific terminology and the ways to cope than new residents who have never experienced a flood, or if flooding occurs infrequently, the memory of past events may not be part of inter-generational cultural knowledge.

We know that individual-level responses to environmental changes are time dependent and heterogeneous, with some individuals coping quickly and others taking one or more generations to adapt. What is true for the adoption of technological innovations (Rogers 2003) is also true for behavioural adaptation to environmental change. While a few individuals may adapt quickly to an environmental shift, a population will take at least one generation to become fully adjusted (Moran 2000). Individuals and households also vary in their capacities to respond: younger adults cope with the change more quickly than elderly adults because it is part of the new experiences and cultural scripts they incorporate as they mature, whereas elders have longer contradictory experiences, set behavioural routines and have more to lose if the change is short term rather than permanent.

In addition, households have different economic levels, thus different capacities to take risks (Cancian 1989). Those at the top of the social ladder have a lot to risk in terms of cost if they change their practices for the short term, and the consequences of a mistake can result in a downward slide in status and wealth. Other community members who aspire to be at the top of the ladder are more eager to take risks with high potential for positive pay-off. Thus, they are more likely to innovate in their strategies, with hopes of racing ahead. Should their risk pay off, the wealthier families may follow to protect their positions, but some will resist change for some time.

One of the challenges to human adaptation to climate change is to understand the scale of the problem: most environmental perception is local rather than global, and is manifested in experience with changes in precipitation and temperature and observation of crop responses to current conditions (see diverse examples in Magistro *et al*. (2001)). Moreover, the changes are likely to occur within a very short time frame, normally 2 or 3 years. At this temporal scale, there is much fluctuation in temperature and precipitation; thus the data are ‘noisy’ and hard to interpret. Local perspective confounds this because within any given watershed or region, people know that properties and households can have different experiences with crop responses, precipitation and temperature.

We need to recognize these relationships, in the Amazon and elsewhere, as complex cross-scale problems (Brondizio *et al*. in review). The literature on human–environment interaction has had a tendency to treat human responses to change at a local, self-contained level, understandably an attractive approach from an analytical perspective, but limited in capturing the linkages and vertical interplay created by a growing functional interdependency of resource use systems and ecosystems (Berkes 2006; Young 2006). Achieving a link between local and global understanding of climate change requires connections within a broad information network, and a trust that the information from both levels is credible (Cash *et al*. 2006). When the national and global forecasts contradict local experience, people resist believing them because they do not match personal understanding and experiences of climate patterns.

Research on human adaptation to climate events has been encouraged by the Climate and Global Change Program of the US National Oceanic and Atmospheric Administration to understand how people respond to weather forecasts. This has resulted in a growing body of evidence for the roles of institutions, culture, social class and perception in how people respond to forecasts (e.g. Nelson & Finan 2000; Orlove *et al*. 2000; Lemos *et al*. 2002; Moran *et al*. 2006). Where robust local institutions exist and are linked to larger institutions, the flow of information is quicker and more effective (Ostrom 2005; Toniolo 2005). Where a connection exists between climate factors and cultural practices, adaptation is likely to be quicker (Magistro *et al*. 2001). Most of the research has identified obstacles to people believing and acting upon the forecasts, which come largely from the mismatch of local experience and national and global forecasts. In the Amazon, research and forecasting models for phenomena like ENSO and climate change, in general, are produced at coarse resolutions and scale: global; continental; and macroregional. Information at these levels is often all that is communicated to rural communities and farmers, given the limitations of, on the one hand, information dissemination systems and, on the other hand, data and models in generating locally precise data to most places on the planet (Pfaff *et al*. 1999; Stern & Easterling 1999).

However, the growing interdependence of resource use systems, commodity markets and climate requires attention to mechanisms operating at larger scales but influencing local-level adaptation. For instance, it is important to understand how farmers engage in collective action and interact with other sectors of the population within the landscape and affected by each other's land-use behaviours, such as the use of fire or management of a watershed. We also need to consider the way farmers receive and interpret information derived from global and national forecasts projecting interannual and seasonal climate patterns or weekly and daily weather information. The media play a central but not always informative role in disseminating this information, particularly in areas such as the Amazon, where the scale tends to be broad, data resolution is coarse and other forms of dissemination are infrequent and have limited reach. A farmer's interpretation and trust of such information depend on the spatial and temporal scales with which it is presented, its relevance to local-level decision making, clarity of language and terminology and even how politicized it appears (e.g. serving particular economic interests).

## 3. Results: household survey data

We found that for most farmers, memories of extended drought tend to decrease significantly after 3 years. Over 50% of the farmers interviewed in 2002 did not remember as significant the ENSO drought of 1997/1998, one of the strongest on record. This helps explain why approximately 40% of the farmers have not changed their land-use behaviours in the face of the strongest ENSO event of the twentieth century. Our household survey data in Pará State document conditions underlying this important finding. Farmers report various impacts of climate anomalies, such as lost productivity in ENSO years (57% in Santarém and 51% in Altamira) and direct economic losses from accidental fire (37% in Santarém and 38% in Altamira). Survey data show the rate of accidental fire triples during ENSO years. However, the reality of most small farmers in the Amazon is a lack of extension services for adoption of new agricultural techniques, and climate information relevant to local level land-use decision is also nearly absent. When asked to rank their priorities, farmers elicit poor infrastructure (e.g. roads/transportation cited as top concern of farmers) and limited access to credit and technology as their main problems. For the two surveyed areas, the proportion of farmers depending on manual labour and simple tools exceeded 95% with only 18% reporting some access to tractors, for instance, to construct ponds and/or open firebreaks. Among other factors, poor infrastructure and the lack of extension services and commercialization support lead to high rates of lot turnover, which has averaged 75% in the two settlements, where over one-third of rural communities have existed less than 20 years. The pressure from capitalized, large farms further accentuates this problem: land buyouts leading to loss of population, which consequently impacts the availability of services (e.g. schools, transportation) in rural communities. Under these circumstances, adaptive decision making is made infinitely more difficult since it disrupts sharing of farming experiences as well as the rise of collective action at the community level.

Although farmers pay attention to climate information provided by the media (cited by over 40% of farmers surveyed), the scale of information (usually for the Amazon region as a whole or state level but no local information) does not motivate changes in local farming behaviour. Less than 5% of farmers surveyed reported receiving specific information about the 1997/1998 ENSO event. In this context, farmers tend to use local methods, such as animal behaviour, plant phenology, testing salt humidity and sharing information (Moran *et al*. 2006). Local weather experts are also recognized, and often cited as reliable, in interpreting environmental signals as indicators of weather and what to expect in seasonal climate patterns. Thus, it is not surprising that over 75% of farmers rely on personal experiences to assess the level and impact of drought or excessive rainfall to make decisions about land use and livelihoods. As a whole, the proportion of farmers relying on the media, non-government organizations (NGOs), government offices, extension services and community groups to assess climate information for land-use decision tends to be less than 10% in both study areas (Moran *et al*. 2006).

The dynamics described above undermine the collective and individual memories of climate events and the way local history of them is transmitted. To most farmers, for instance, memories of extended drought tend to decrease significantly after 3 years, and only dramatic events or personal experiences are still remembered by a small percentage of the population. However, when interviewed in 2001, although not necessarily changing land-use behaviour over 85% of farmers reported some action concerning prevention of accidental fires, ranging from firebreaks to informing neighbours about times and locations of intended burnings. When possible, they compensate the impact of drought by tapping into family savings (e.g. cash withdrawals, selling cattle) to cope with lost productivity. Those who changed their land-use behaviours did so in small steps. Adaptation strategies to drought included delays in burning and planting, additional opening of firebreaks and seeking more communication with neighbours regarding agreements about when, where and how to burn.

## 4. Conditions undermining adaptation to climate change in the Amazon

One of the best documented challenges to climate change is human response to extreme floods and droughts. From early literature on natural hazards (White 1974), the findings were startling at the time and are still overlooked today. Natural hazard scientists showed how these events were reinterpreted within 3 years of occurrence if the event did not recur, such as a severe drought being interpreted and considered a ‘dry spell’. Floods were also largely forgotten within 3 years, particularly if reconstruction was successful. The conclusions of many studies in the 1960s and 1970s were that people would rather live with risk than uncertainty. The latter immobilizes individuals who keep waiting for disaster to hit again. After approximately 3 years, the individuals would rather put away the uncertainty and deal with risk. We can, for example, hypothesize that adaptation to El Niño and La Niña events will begin to occur as they increase in frequency and are more salient in the public imagination and information system. The frequency of these events has gone from 20 to 10 years and possibly 5 years (Moran *et al*. 2006), but the high rate of replacement of rural populations in our study challenges collective learning from the previous experiences and preventive actions.

Rural populations are highly variable in their position in society; have varying degrees of knowledge and access to resources such as land, labour and capital; and have varying degrees of institutional self-organization that affect their ability to adapt to changing conditions (Netting 1993; Ostrom 2005). At a local level, numerous factors influence this adaptive capacity: time in the region; knowledge of the environment; density of social networks; quality and quantity of people's economic portfolios; available land-use technology that people can afford; memories of past events and how they are applied in the present; and actual social relations and memory of them. We know that long-time residence in a region is centrally important, as studies have shown that people learn about their environment over time, their knowledge becomes culturally embedded and it is highly precise in extent and detail (e.g. Conklin 1961, 1969; Berlin 1992; Orlove *et al*. 2000). The difference between long-term residents of a region and colonists in the Amazon has always been a subject of interest (Moran 1975, 1989; Brondizio 2004, 2006, 2007). Upon arrival, colonists are superficial in classifying their physical environment, and that it is based on their knowledge of past locations, whereas natives have a vertically complex system of classification embodying many variables as a result of cumulative knowledge of environmental information (Moran 1975; Behrens 1989; Brondizio & Neves 1997).

In this context, the rate of lot turnover in the Amazon and continuous formation and transformation of rural communities undermine the ability of rural populations to learn, share and develop forms of individual adaptation and collective action to cope with climate change. The density of social networks plays a key role in adaptation because social networks embody community knowledge that contributes to assessing the best adaptive paths consistent with community integrity. This ensures that change is consistent with community values, but it can also retard adaptation, especially during the periods of rapid change. Conflict can arise between individuals who want to change and adjust and those who wield more power in the network and resist adaptation in fear of threats to the local system of authority. In general, however, communities with dense social networks have an advantage in group response. For instance, kinship systems and customary rules among ‘traditional communities’ in the Santarém-Belterra region minimize transaction costs and motivate forms of collective action to avoid the spread of accidental fire during years of extended drought, which contrasts with neighbouring, recently colonized communities that have higher transaction costs for organization and where the frequency and extent of accidental fire are significantly higher (Toniolo 2005).

At a regional scale, the spatial distribution of rural population is important, as this creates differential conditions for acquiring knowledge and for moving people and resources across a changing landscape; differences in physical infrastructure give farmers different opportunities to adjust their behaviours; access to respected extension services can shorten the adjustment time, but small farmers worldwide tend to have less access than large farmers. Because small farmers rarely occupy prime lands and locations, they are harder to serve than large farmers, and the pay-off to the agencies that serve them is smaller. Farmers in both study areas reported that some extension agents actually requested help with fuel cost to visit farms and often had no available transportation. The effective activities of institutional networks such as churches, NGOs and cooperatives can reduce uncertainties for farmers, or augment them if they promote certain kinds of change but are unable to provide the necessary infrastructure. At national and global scales, the challenge is how to create monitoring systems and flows of information that can serve the needs of the rural sector, including small farmers. Given the above constraints, particularly location and infrastructure limitations faced by small farmers, it is not surprising that dissemination, to them, of information relevant to climatic change has been difficult or meaningless from a practical perspective (Pfaff *et al*. 1999; Costa 2006). The effectiveness of disseminating climate information at different levels and to different groups is challenged by its scale and resolution, its perceived immediate relevance, differences in knowledge systems, differences in language and terminology and the legitimacy and credibility of institutions involved in the process. In this sense, there is a cultural divide between the ability of farmers to understand the lexicon and the symbols of global and regional climate forecasting and the ability of scientists to understand and value the knowledge and perceptions farmers have of their environment and climate (Agrawal 1995).

This impasse can be broken in a variety of ways. First, more micromet stations are needed to collect data that are locally relevant and that can inform climate models and forecasts at a finer spatial resolution. Second, agricultural extension organizations working with farm organizations need to pass this information to farmers in a timely fashion, such as through radio and television programmes. Third, farmer organizations and communities should be encouraged to be participants in the production of scientific and reliable information, rather than be treated only as consumers of such information. It is unlikely that federal, state or local governments have the budget flexibility to put a dense network of micromet stations for an area as large as the Amazon in the near future. Yet, farmers need this kind of information now, as climate change is taking place at a pace that is outpacing the ability of governments to generate timely information. Farmers, and schools in rural communities, could work together to implement micromet stations at low cost (following something akin to the GLOBE approach), and engage students in monitoring climate changes in temperature and precipitation as part of their school work and learning about science.^1^

We call attention to the importance of nesting our understanding of individual environmental cognition and behaviour into larger social, cultural and environmental contexts. This includes considering the role of climate monitoring, forecasting and dissemination systems that are framing for society and policy makers the connections between the climate change and the fate of the Amazon.

Several processes mediate the perception of change in environment and climate and differ at individual, household, community and regional scales. Perhaps the most important among them are various social conditions that differentiate between individuals and communities in their adaptability to climatic change, which, in the Amazon, require new approaches to understanding adaptation.

## Figures and Tables

**Table 1:** Vulnerability of Amazonian small farmers to some environmental conditions associated with climate change.

extended drought	extreme variation in flood level	accidental fire
planting time and risk of ‘seasonal trap’	residency pattern	conflict with neighbours
loss of crops and productivity	access fishing and planting grounds	risk invested capital
flammability of land cover	transportation and access to market	flammability of land cover
water quantity and quality	water quantity and quality	loss of biodiversity
infectious and non-infectious diseases	infectious diseases	non-infectious diseases

## Supplementary Material

El Niño/climate kit for farmers and rural communities El Niño/climate kit for farmers and rural communities

## Endnote

1 Electronic supplementary material illustrates an ENSO/Rainfall kit prepared for farmers in both study areas. The authors conducted several workshops with farmers to share project results and distribute a guide and rainfall gauge to farmers and local communities aiming at providing initial training on the use of rain gauge and rainfall data recording and evaluation.
