# Supplementary material for: Human dimensions of climate change: the vulnerability of small farmers in the Amazon
Source: Philos Trans R Soc Lond B Biol Sci. 2008 Feb 11;363(1498):1803–9. doi: 10.1098/rstb.2007.0025 (PMC2374891; doi:10.1098/rstb.2007.0025)

Kit do(a) agricultor(a) para coleta de dados pluviométricos e indicador de possíveis anos do *El Niño* e seca

Produzido pelo Centro Antropológico de Pesquisa e Treinamento em Mudanças Ambientais Globais (ACT)
Student Building 331
Indiana University
Bloomington, IN 47405, EUA

Junho de 2003

Tel: (812)855-6181
Fax: (812)855-3000
Email: act@indiana.edu
Web: http://www.indiana.edu/~act

Índice

Introdução..1

Instalação e Uso do Seu Pluviômetro.....................2

Compreensão das Chuvas......................................3

Formulário de Chuvas Mensais..............................7

Formulário Gráfico (diariamente para um mês)...19

Formulário Gráfico (media mensal para um ano).20

Reconhecimentos

Esta pesquisa foi viabilizada pelo apoio financeiro da NOAA, Agencia de Pesquisa Atmosférica e Oceânica Nacional dos E.U.A. ao projeto "Estratégias Humanas relacionadas com El Niño nas Florestas da Amazônia". Esta pesquisa também foi possível pelos acordos colaborativos com várias organizações e indivíduos listados abaixo (mas não se limita somente aos listados abaixo):

Indiana University
http://www.indiana.edu

The Anthropological Center for Training and Research on Global Environmental Change (ACT)
http://www.indiana.edu/~act

The Center for the Study of Institutions, Population, and Environmental Change (CIPEC)
http://www.cipec.org

The National Oceanic and Atmospheric Administration (NOAA)
http://www.noaa.gov

Empresa Brasileira de Pesquisa Agropecuária (EMBRAPA)
http://www.embrapa.br/

EMBRAPA/CPATU
http://www.cpatu.embrapa.br/
Campus: Altamira, Medicilândia, Belterra, e Santarém

Instituto de Pesquisa Ambiental da Amazônia (IPAM)
http://www.ipam.org.br/

O Experimento de Grande Escala da Biosfera-Atmosfera na Amazônia (LBA)
http://www.cptec.inpe.br/lba/

Escritórios EMATER de Santarém, Belterra, Altamira, Brasil Novo, and Medicilândia, Estado do Pará.
http://www.emater.pa.gov.br

Instituto Cultural Boanerges Sena (ICBS)

Gostaríamos de agradecer a contribuicao dos senhores António Manzi, Ryan Adams, Eliazar Brait na preparacao deste guia e de diversas. Também de agradecer os dados pluviométricos fornecidos pelo Sr. Emar Franco da Cunha e o apoio logístico prestado pelo escritório de Santarém do Programa LBA.

Introdução

Este documento, o "Kit do agricultor para coleta de dados pluviométricos e indicador de possíveis anos de *El Niño*", foi criado como parte de um projeto de pesquisa com o título de "Estratégias Humanas relacionadas com El Niño nas Florestas da Amazônia", e foi financiada pela NOAA. Há três anos as nossas equipes de pesquisa têm coletado informações de várias fontes nas regiões de Altamira e Santarém, estado do Pará, Brasil. Temos entrevistado pesquisadores, agentes governamentais, lideres de comunidades e extensionistas e também temos avaliado o uso da terra de dezenas de unidades domésticas agrárias nas duas regiões. Temos desenvolvido este "kit" para dar um subsídio aos produtores da região que possa aumentar a qualidade e quantidade de informações sobre a precipitação a nível local. Desta forma os produtores mesmos poderão ter uma base para tomar suas decisões agrícolas com maior confiança. O risco de perdas econômicas com fogo acidental (por exemplo, cercas, áreas de plantações e floresta) tem aumentado com o tempo devido a maior área cultivada, e a eventos climáticos como o El Niño, nos quais ocorrem estiagens prolongadas. A probabilidade do fogo escapar das áreas preparadas, para áreas vizinhas, cria uma situação às vezes difícil.

Serão entregues a cada um de vocês um pluviômetro para medição das chuvas. Neste documento explicamos como instalar o pluviômetro, como e quando coletar a informação, e como manter os dados de uma forma que facilite seu manejo agrícola com base no padrão de chuvas da sua propriedade. Tal padrão também será relevante a seus vizinhos, assim você poderá ser uma forte de informação para eles também.

Este documento apresenta várias partes. A próxima seção de "Instalação e Uso do seu Pluviômetro" descreve o pluviômetro, sua instalação correta, como lê-lo, e como anotar corretamente os dados de chuvas. A seção seguinte, "Compreendendo as Chuvas", dá exemplos de padrões de chuvas para sua área. Os exemplos são dados num modelo de como a sua própria informação pluviométrica deveria ser anotada e graficamente representada, e também como fazer uma comparação para que você possa estimar (avaliar) a variabilidade do clima local e ver se o padrão que está se vendo indica um padrão normal (como em 1994), um padrão El Niño (estiagem, como em 1997 e 1998), ou um padrão de maior chuva (como ocorreu em 2000 e 2001). A porção

final deste documento fornece formulários de coleta de dados de chuvas (para o primeiro ano) e gráficos formatados para demonstrar graficamente as chuvas diárias no período mensal e o total de chuvas mensais no período anual.

Instalação e Uso do Seu Pluviômetro

Esta seção descreve o seu pluviômetro, a sua instalação correta, como lê-lo, e também como anotar as chuvas observadas. O pluviômetro oferecido com este "kit" é uma peça de plástico transparente única (veja a Fig. 1). O pluviômetro esta demarcado milímetros, com intervalos de 1mm e 10 mm. Há duas flanges que têm buracos para poder parafusar (afixar) o pluviômetro a um poste (pilar/estação). Verifique se você montou o nível do pluviômetro corretamente e, se possível, ele deveria ser colocado numa área aberta. O pluviômetro deveria ser montado corretamente, sendo afixado verticalmente num poste (pilar/estação) de 1.5 a 2 metros acima do solo (como indicado nas instruções que vêm com o pluviômetro).

Ao ler e anotar a quantidade de chuvas observadas no seu pluviômetro é importante ser o mais preciso possível, especialmente se você for compartilhar os seus dados com outras pessoas da comunidade e região. As informações a seguir irão lhe ajudar a ser o mais preciso possível. Quando o pluviômetro começa a encher com as chuvas, você vai perceber que a superfície da água é curva. Esta superfície é chamada de menisco e é formada pela tensão da superfície da água com os lados do pluviômetro. Você deveria anotar o total de precipitação das últimas 24 horas com os dados da base do menisco (superfície mais baixa da curva do menisco). Depois de uma chuva e preferível ir e fazer a anotação o mais rápido possível porque se o sol sair, poderá evaporar a água do pluviômetro e aparentar não ter chovido. Se chover mais de uma vez, seria preferível anotar o nível da água após a cada chuva, a menos que o sol não sair no caso e preferível esperar pelas 24 horas. No normal, e preferível escolher um horário certo e coletar as informações nesse horário, com a exceção de chuvas seguidas por sol forte de várias horas. É sempre preferível instalar o pluviômetro em um lugar conveniente para os membros da família poder coletar os dados.

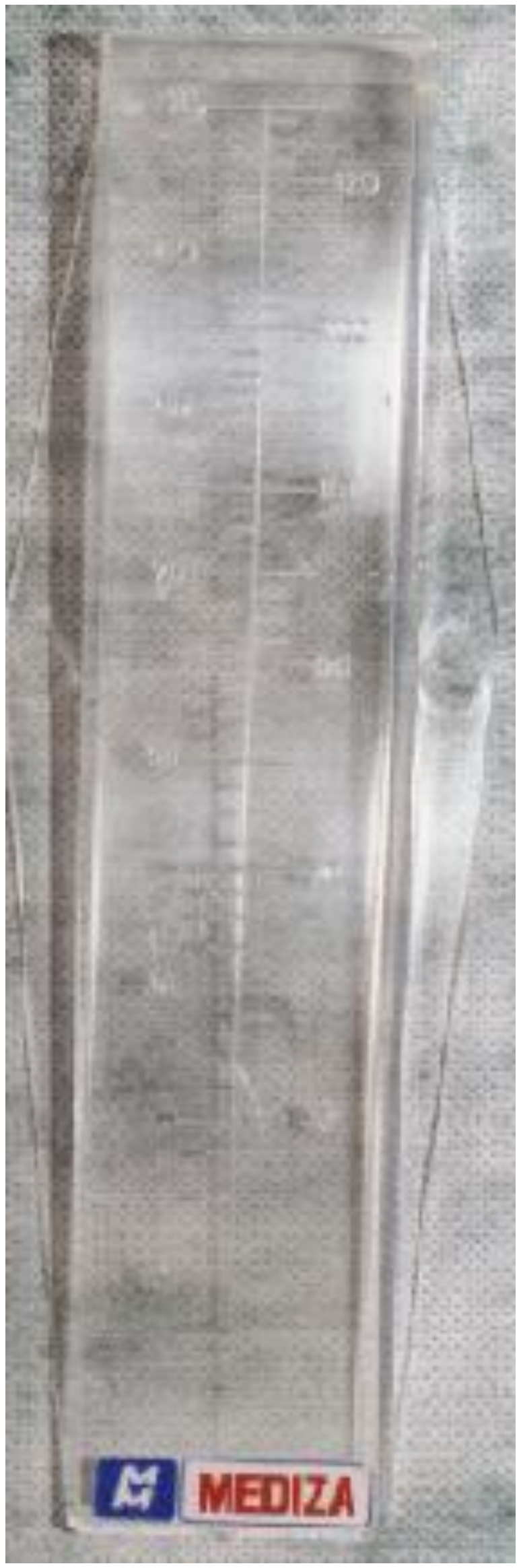

Figura 1, Pluviômetro

Nos formulários de coleta de dados pluviométricos, incluídos neste kit, você precisará anotar o número total de chuvas observadas em milímetros. O formulário de coleta de dados de chuvas tem vários tipos de informações diferentes que são necessárias para que seus dados de observação de chuvas sejam mantidos (e possivelmente compartilhados). Você vai perceber que o formulário das chuvas tem um espaço para anotar a hora usual de coleta de dados assim como a hora real que você coletou os dados. É muito importante que você colete os dados da chuva no mesmo horário todos os dias e se você não puder fazer isso, anote quando os dados de chuvas foram realmente coletados (hora real).

Compreensão das Chuvas

Esta seção dá exemplos de padrões de chuvas para as duas áreas pesquisadas. Há exemplos de como as suas informações sobre chuvas deveriam ser anotadas e graficamente representadas, e também há exemplos de comparação para que você possa avaliar (estimar) a variabilidade do clima local. Para as duas áreas estudadas temos bancos de dados diferentes. Para Altamira temos dados de chuvas de 1972 a 1993. Nós não temos dados pluviométricos que sejam mais recentes para Altamira neste momento, e, por isso, não podemos prover uma tabela de dados de chuvas para o

período do El Niño mais recente na região. No entanto, o período de vinte anos que nós representamos graficamente oferece exemplos de padrões de dados pluviométricos para a região de Altamira. Nossa pesquisa indicou que os anos de 1981/1982 e 1992 foram anos secos. Em ambos os anos houve períodos que foram significantemente mais secos do que o normal.

Os dados de chuvas para Santarém, de 1980 a 2000, são fornecidos. Também fornecemos uma tabela do período dos cinco anos mais recentes para Santarém. Os anos de 1997 e 1998 foram particularmente secos na região de Santarém, e estes anos coincidem com o fenômeno do El Niño. Em ambos os anos houve cinco ou menos meses com chuvas maiores de 100mm. Na região de Santarém, os meses de setembro a dezembro são tipicamente mais secos do que o resto do ano. Quando os meses de Novembro e Dezembro são particularmente secos, com pouca chuva ou sem chuvas, é possível observar prováveis efeitos do El Niño, e, mais precauções são necessárias para evitar que o fogo se espalhe. Na região de Altamira é mais difícil avaliar os impactos do El Niño porque até em meses secos, quase sempre há chuvas. Os poucos meses no gráfico que não há dados de chuvas são potencialmente erros na anotação das chuvas, ao invés de meses que não houve chuvas. Os dados sugerem que o fenômeno do El Niño em 1982 causou impactos (efeitos) extensivos no período de dois anos, de 1982 a 1983, com níveis de chuvas menores do que a média, até nos meses mais chuvosos. No entanto, o ano de 1984 recuperou rapidamente a escassez de chuvas dos anos anteriores. Em comparação com a região de Santarém, o impacto do El Niño em Altamira parece ser menor, mas isso não significa que precauções contra incêndios (fogos) não devem ser tomadas, principalmente nos meses mais secos. Esperamos que este kit ajude neste planejamento, fornecendo meios para que você possa manter/anotar os dados de chuva e de variabilidade do clima na sua fazenda.

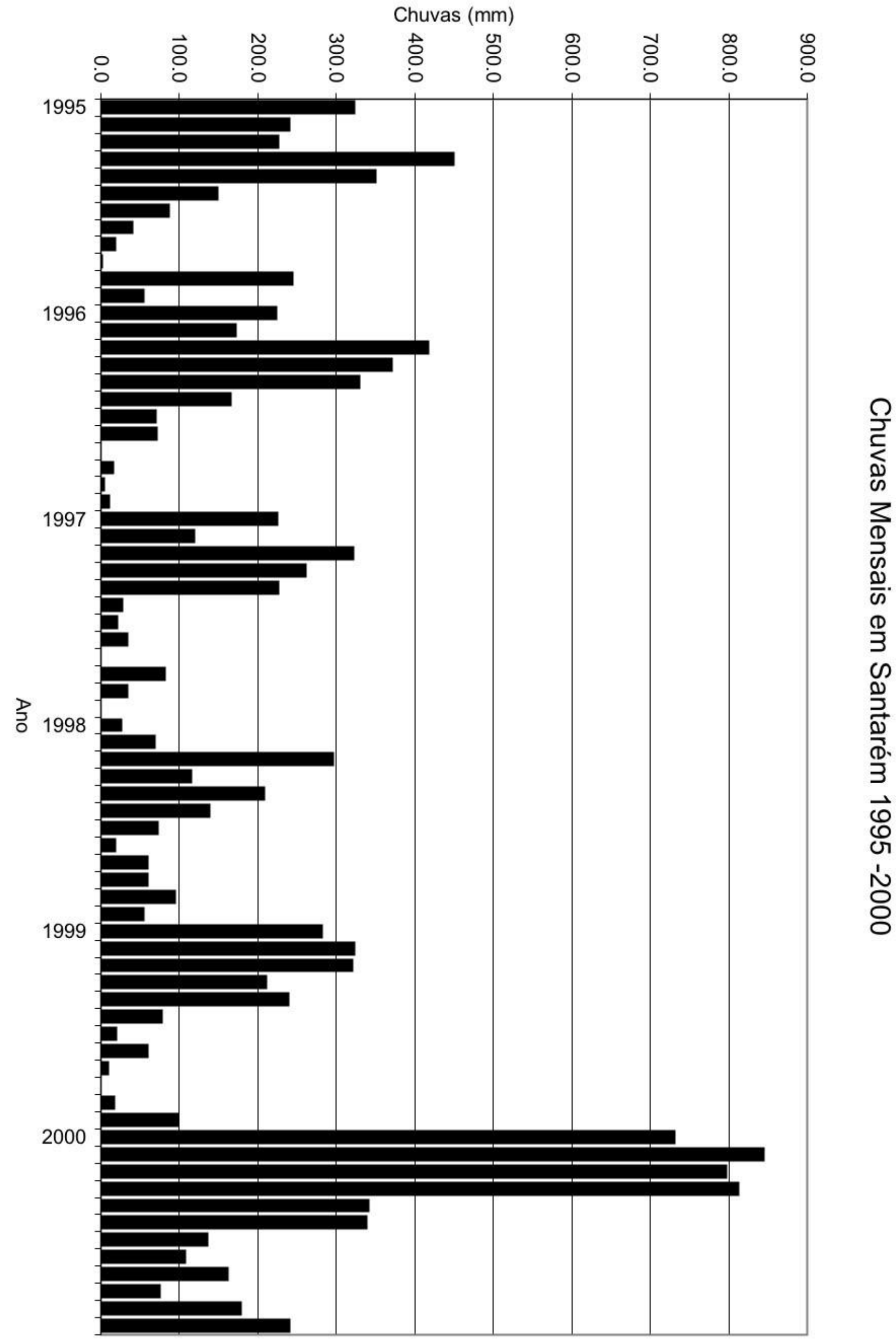
Chuvas Mensais em Santarém 1995 -2000
Chuvas (mm)
0.0
100.0
200.0
300.0
400.0
500.0
600.0
700.0
800.0
900.0
1995
1996
1997
1998
1999
2000
Ano

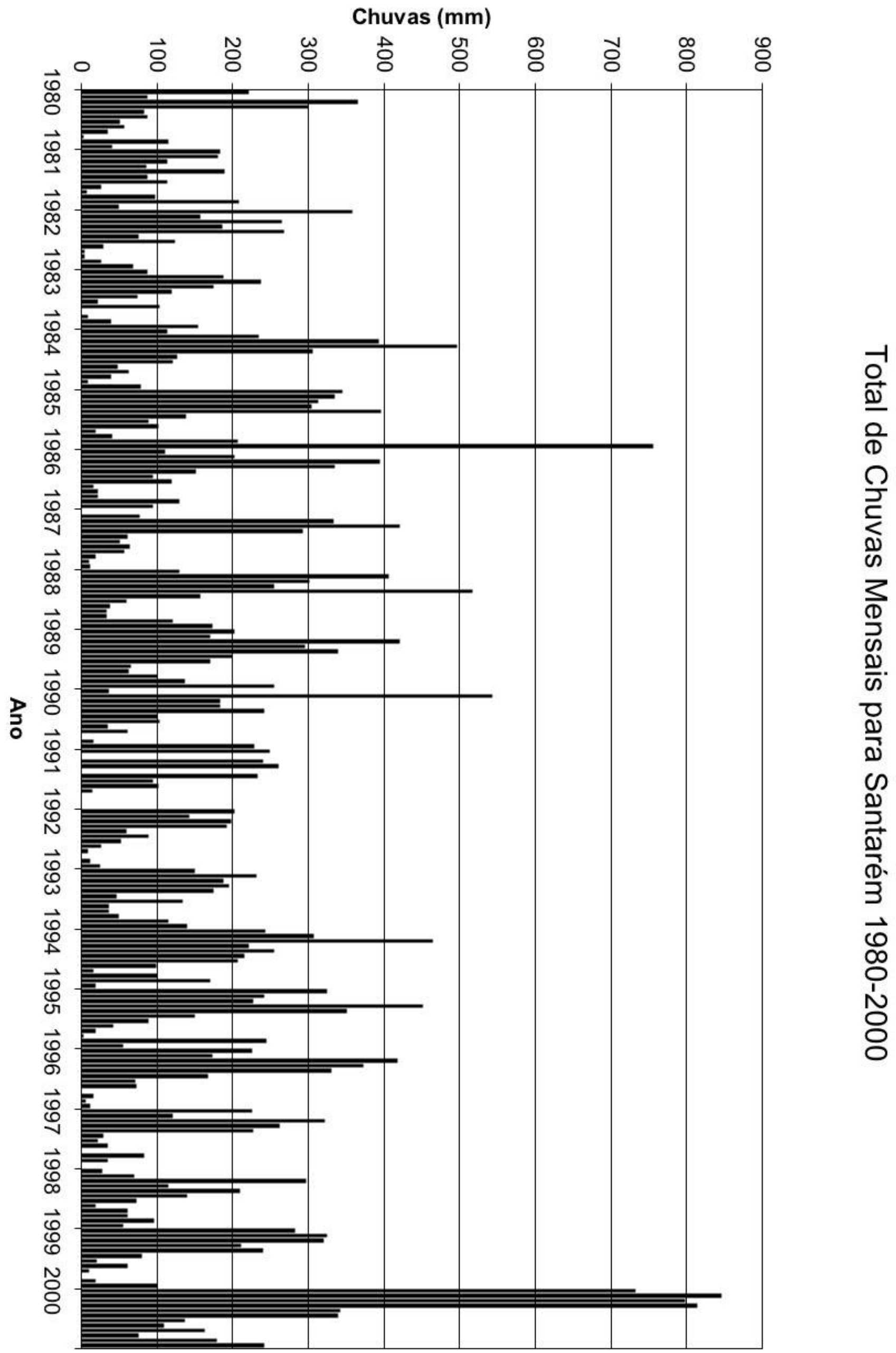
Total de Chuvas Mensais para Santarém 1980-2000
Chuvas (mm)
0
100
200
300
400
500
600
700
800
900
1980
1981
1982
1983
1984
1985
1986
1987
1988
1989
1990
1991
1992
1993
1994
1995
1996
1997
1998
1999
2000
Ano

Total de Chuvas Mensais para Altamira 1972-1992

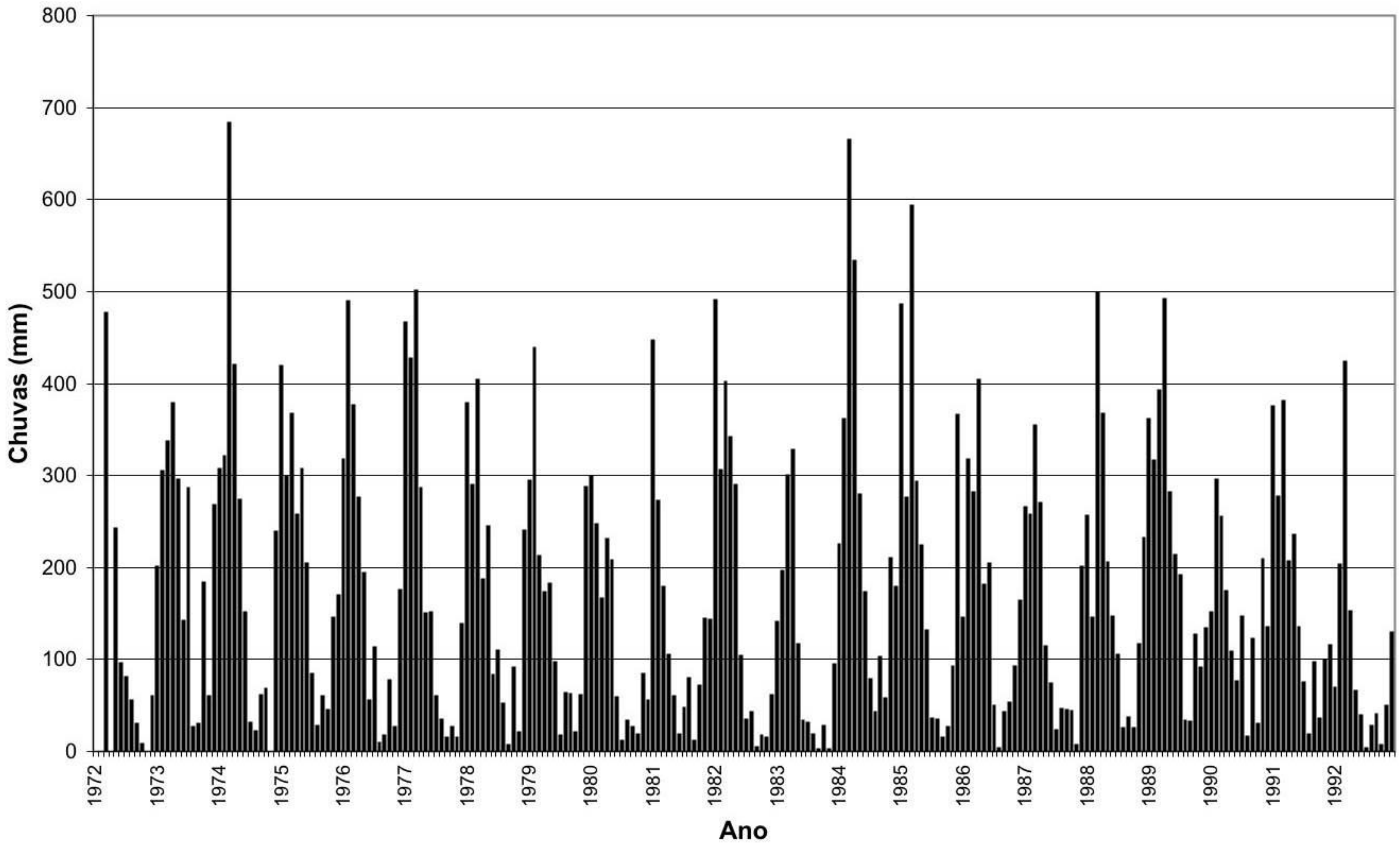

Formulário de Chuvas Mensais

Somente um ano do formulário de chuvas mensais está incluído aqui. Por favor, duplique (copie) esta página para anotar mais anos ou anote os dados de forma similar num caderno comum.

Nome da pessoa anotando os dados de chuvas: ___________________________
Município/Endereço: _____________________________________

Mês:__________ Ano: _________ Hora de Observação usual: ________ Manhã Tarde (circule um)

**Dia**	**Hora de Observação Real**	**Chuvas (mm)**	**Notas** (por exemplo – se o tempo estiver diferente do normal ou se alguma outra pessoa estiver anotando os dados de chuvas)
1			
2			
3			
4			
5			
6			
7			
8			
9			
10			
11			
12			
13			
14			
15			
16			
17			
18			
19			
20			
21			
22			
23			
24			
25			
26			
27			
28			
29			
30			
31			
	**Total:**		
	**Total/No. de dias (divisão)**		Esta é a sua média diária no mês

Nome da pessoa anotando os dados de chuvas: ___________________________

Município/Endereço: _____________________________________

Mês:__________ Ano: _________ Hora de Observação usual: ________ Manhã Tarde (circule um)

Dia	Hora de Observação Real	Chuvas (mm)	**Notas** (por exemplo – se o tempo estiver diferente do normal ou se alguma outra pessoa estiver anotando os dados de chuvas)
1			
2			
3			
4			
5			
6			
7			
8			
9			
10			
11			
12			
13			
14			
15			
16			
17			
18			
19			
20			
21			
22			
23			
24			
25			
26			
27			
28			
29			
30			
31			
	**Total:**		
	**Total/No. de dias (divisão)**		Esta é a sua média diária no mês

Nome da pessoa anotando os dados de chuvas: ___________________________

Município/Endereço: _____________________________________

Mês:__________ Ano: _________ Hora de Observação usual: ________ Manhã Tarde (circule um)

Dia	Hora de Observação Real	Chuvas (mm)	**Notas** (por exemplo – se o tempo estiver diferente do normal ou se alguma outra pessoa estiver anotando os dados de chuvas)
1			
2			
3			
4			
5			
6			
7			
8			
9			
10			
11			
12			
13			
14			
15			
16			
17			
18			
19			
20			
21			
22			
23			
24			
25			
26			
27			
28			
29			
30			
31			
	**Total:**		
	**Total/No. de dias (divisão)**		Esta é a sua média diária no mês

Nome da pessoa anotando os dados de chuvas: ___________________________

Município/Endereço: _____________________________________

Mês:__________ Ano: _________ Hora de Observação usual: ________Manhã Tarde (circule um)

**Dia**	**Hora de Observação Real**	**Chuvas (mm)**	**Notas** (por exemplo – se o tempo estiver diferente do normal ou se alguma outra pessoa estiver anotando os dados de chuvas)
1			
2			
3			
4			
5			
6			
7			
8			
9			
10			
11			
12			
13			
14			
15			
16			
17			
18			
19			
20			
21			
22			
23			
24			
25			
26			
27			
28			
29			
30			
31			
	**Total:**		
	**Total/No. de dias (divisão)**		Esta é a sua média diária no mês

Nome da pessoa anotando os dados de chuvas: ___________________________

Município/Endereço: _____________________________________

Mês:__________ Ano: _________ Hora de Observação usual: ________ Manhã Tarde (circule um)

Dia	Hora de Observação Real	Chuvas (mm)	**Notas** (por exemplo – se o tempo estiver diferente do normal ou se alguma outra pessoa estiver anotando os dados de chuvas)
1			
2			
3			
4			
5			
6			
7			
8			
9			
10			
11			
12			
13			
14			
15			
16			
17			
18			
19			
20			
21			
22			
23			
24			
25			
26			
27			
28			
29			
30			
31			
	**Total:**		
	**Total/No. de dias (divisão)**		Esta é a sua média diária no mês

Nome da pessoa anotando os dados de chuvas: ___________________________

Município/Endereço: _____________________________________

Mês:__________ Ano: _________ Hora de Observação usual: ________ Manhã Tarde (circule um)

Dia	Hora de Observação Real	Chuvas (mm)	**Notas** (por exemplo – se o tempo estiver diferente do normal ou se alguma outra pessoa estiver anotando os dados de chuvas)
1			
2			
3			
4			
5			
6			
7			
8			
9			
10			
11			
12			
13			
14			
15			
16			
17			
18			
19			
20			
21			
22			
23			
24			
25			
26			
27			
28			
29			
30			
31			
	**Total:**		
	**Total/No. de dias (divisão)**		Esta é a sua média diária no mês

Nome da pessoa anotando os dados de chuvas: ___________________________

Município/Endereço: _____________________________________

Mês:__________ Ano: _________ Hora de Observação usual: ________ Manhã Tarde (circule um)

Dia	Hora de Observação Real	Chuvas (mm)	**Notas** (por exemplo – se o tempo estiver diferente do normal ou se alguma outra pessoa estiver anotando os dados de chuvas)
1			
2			
3			
4			
5			
6			
7			
8			
9			
10			
11			
12			
13			
14			
15			
16			
17			
18			
19			
20			
21			
22			
23			
24			
25			
26			
27			
28			
29			
30			
31			
	**Total:**		
	**Total/No. de dias (divisão)**		Esta é a sua média diária no mês

Nome da pessoa anotando os dados de chuvas: ___________________________

Município/Endereço: _____________________________________

Mês:__________ Ano: _________ Hora de Observação usual: ________ Manhã Tarde (circule um)

Dia	Hora de Observação Real	Chuvas (mm)	**Notas** (por exemplo – se o tempo estiver diferente do normal ou se alguma outra pessoa estiver anotando os dados de chuvas)
1			
2			
3			
4			
5			
6			
7			
8			
9			
10			
11			
12			
13			
14			
15			
16			
17			
18			
19			
20			
21			
22			
23			
24			
25			
26			
27			
28			
29			
30			
31			
	**Total:**		
	**Total/No. de dias (divisão)**		Esta é a sua média diária no mês

Nome da pessoa anotando os dados de chuvas: __________________________

Município/Endereço: ___________________________________

Mês:_________ Ano: ________ Hora de Observação usual: _______ Manhã Tarde (circule um)

Dia	Hora de Observação Real	Chuvas (mm)	**Notas** (por exemplo – se o tempo estiver diferente do normal ou se alguma outra pessoa estiver anotando os dados de chuvas)
1			
2			
3			
4			
5			
6			
7			
8			
9			
10			
11			
12			
13			
14			
15			
16			
17			
18			
19			
20			
21			
22			
23			
24			
25			
26			
27			
28			
29			
30			
31			
	**Total:**		
	**Total/No. de dias (divisão)**		Esta é a sua média diária no mês

Nome da pessoa anotando os dados de chuvas: ___________________________

Município/Endereço: _____________________________________

Mês:__________ Ano: _________ Hora de Observação usual: ________ Manhã Tarde (circule um)

Dia	Hora de Observação Real	Chuvas (mm)	Notas (por exemplo – se o tempo estiver diferente do normal ou se alguma outra pessoa estiver anotando os dados de chuvas)
1			
2			
3			
4			
5			
6			
7			
8			
9			
10			
11			
12			
13			
14			
15			
16			
17			
18			
19			
20			
21			
22			
23			
24			
25			
26			
27			
28			
29			
30			
31			
	**Total:**		
	**Total/No. de dias (divisão)**		Esta é a sua média diária no mês

Nome da pessoa anotando os dados de chuvas: ___________________________

Município/Endereço: _____________________________________

Mês:__________ Ano: _________ Hora de Observação usual: ________ Manhã Tarde (circule um)

Dia	Hora de Observação Real	Chuvas (mm)	**Notas** (por exemplo – se o tempo estiver diferente do normal ou se alguma outra pessoa estiver anotando os dados de chuvas)
1			
2			
3			
4			
5			
6			
7			
8			
9			
10			
11			
12			
13			
14			
15			
16			
17			
18			
19			
20			
21			
22			
23			
24			
25			
26			
27			
28			
29			
30			
31			
	**Total:**		
	**Total/No. de dias (divisão)**		Esta é a sua média diária no mês

Nome da pessoa anotando os dados de chuvas: ___________________________

Município/Endereço: _____________________________________

Mês:__________ Ano: _________ Hora de Observação usual: ________ Manhã Tarde (circule um)

**Dia**	**Hora de Observação Real**	**Chuvas (mm)**	**Notas** (por exemplo – se o tempo estiver diferente do normal ou se alguma outra pessoa estiver anotando os dados de chuvas)
1			
2			
3			
4			
5			
6			
7			
8			
9			
10			
11			
12			
13			
14			
15			
16			
17			
18			
19			
20			
21			
22			
23			
24			
25			
26			
27			
28			
29			
30			
31			
	**Total:**		
	**Total/No. de dias (divisão)**		Esta é a sua média diária no mês

Formulário Gráfico (diariamente – Um Mês)

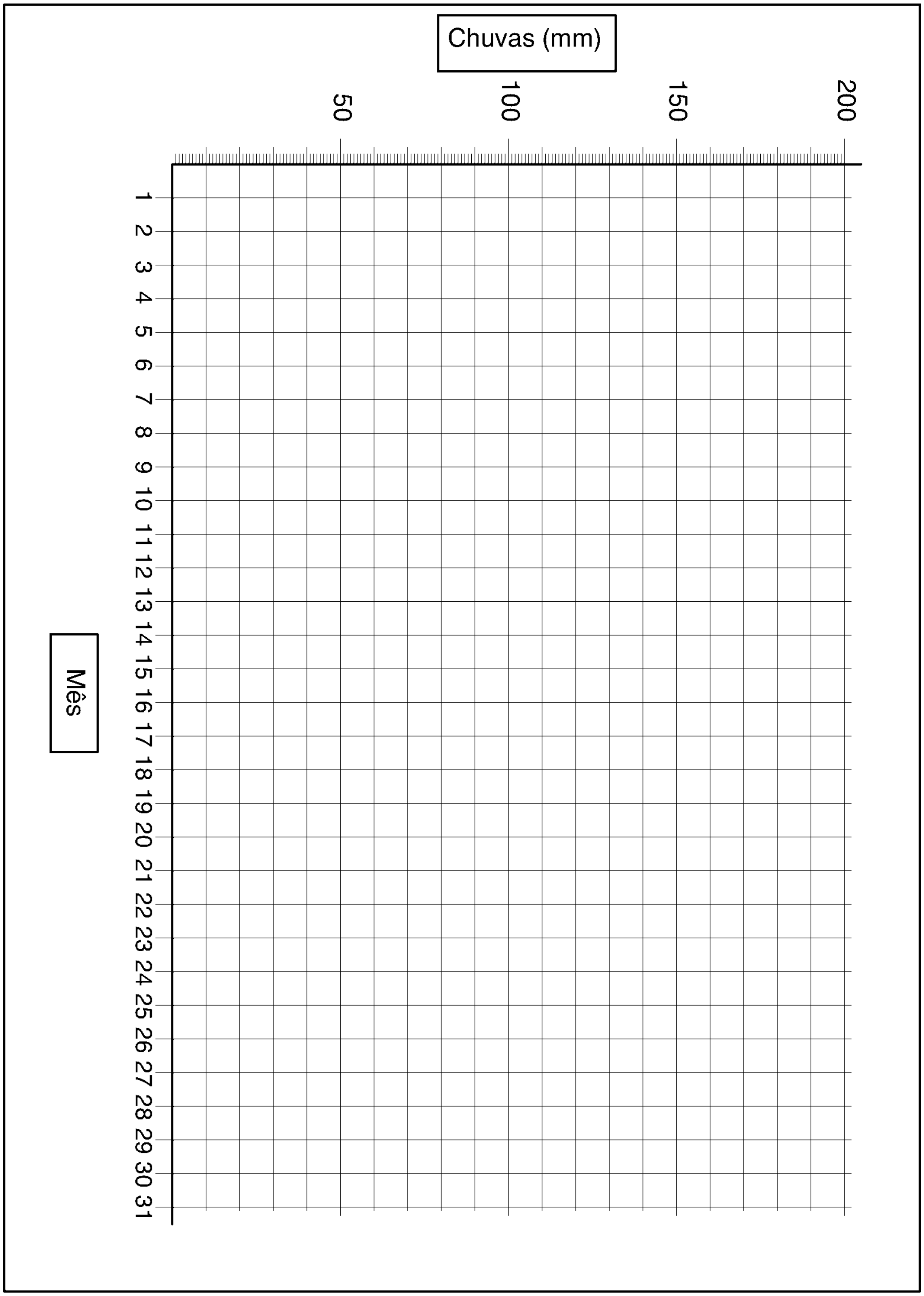

Formulário Gráfico (Um Ano – Média Mensal)

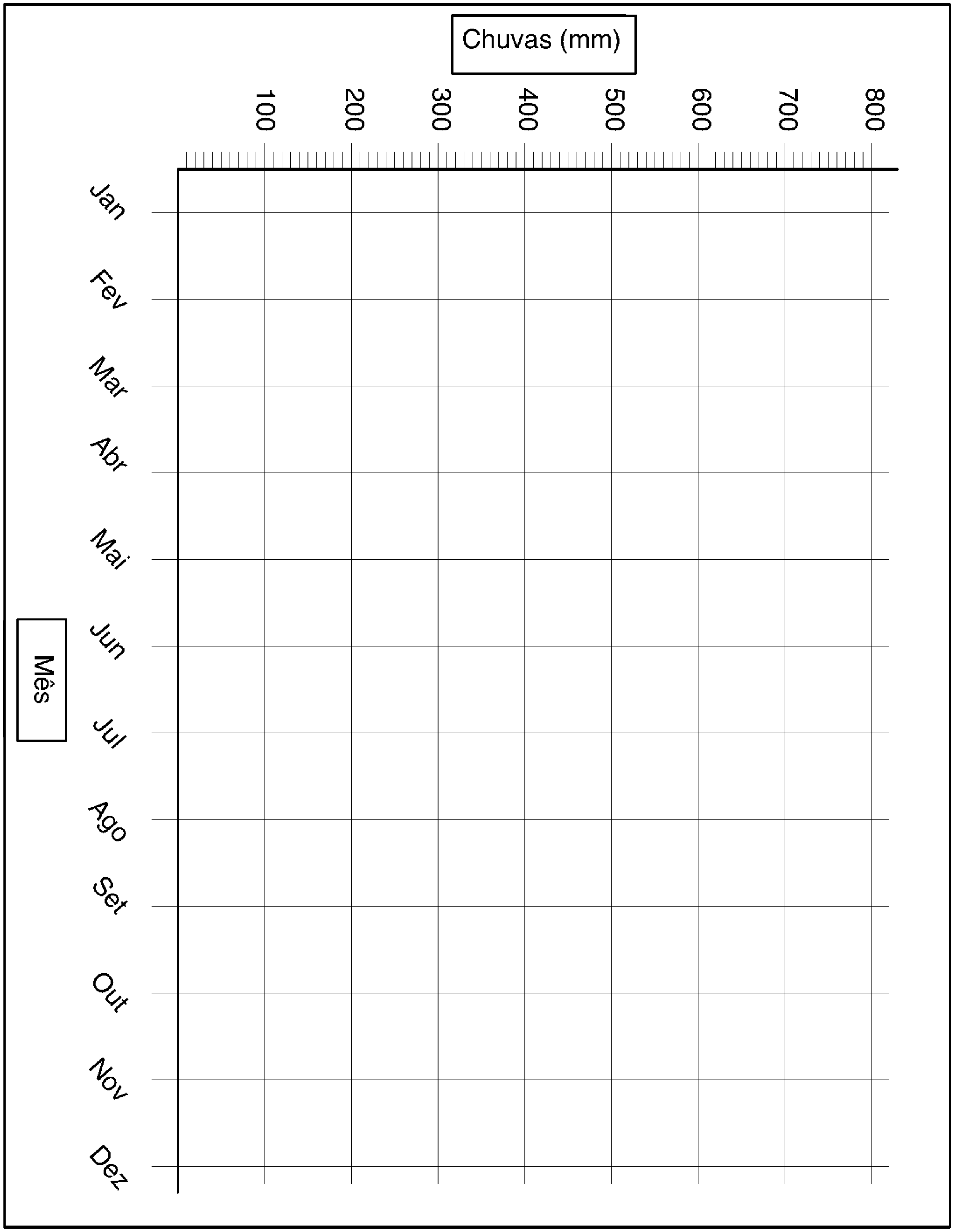

Supplement: Supplementary file 1 [file rstb20070025s02.pdf]
